# Neuralgia

**Published:** 1877-04

**Authors:** M. Holland


### NEURALGIA.

BY M. HOLLAND, M. D.

*A Thesis presented to the Faculty of the Dental College of the University of Michigan, for the degree of D. D. S.*

Neuralgia:—A symptom of some disease or local lesion. It may be caused by thermal changes.

Persons riding in the wind, especially when it is crispy, sometimes have a twinge of pain in the supra-orbital region, and on coming near the stove, it may become more apparent. "Cooks" very often are troubled with neuralgic pains

A fireman once came with a severe pain extending from the supra-orbital notch to the temple. I applied aconite liniment, with internal administration of quinine in large doses; the next day the pain not decreasing in the least, a hypodermic injection of morphia was used and quinine increased until cinchonism (ringing or buzzing of the head) was produced, but in spite of the treatment, the neuralgia lasted over a week, then disappearing as quickly as it came, and not appearing again for four months.

It then made its appearance in the same place as before, and the same treatment being used, the neuralgia lasted but three days, then passed off, and has not returned since.

There was a periodicity of the pain in this case which I should mention; the pain being more severe from eleven p. m. to three a. m. Neuralgia is sometimes a precuasor of marked fever or intermittent. It is said that malaria is one of the principal causes, as almost all of the neuralgic cases complain of the pain being more severe at some hour of the day than others, and this generally occurs in the early morning.

And those people living in low malarious districts are more prone to the disease than those in higher districts.

Probably the most frequent immediate cause, is impingement upon the nerve structure; as by exostosis, which sometimes will cause neuralgia of the side of the face, and can be relieved only by the extraction of the tooth; and a case is reported of a lady who had suffered with severe pain, supposed to proceed from the first lower molar on the right side. This continued for nearly a year, and the patient growing very much emaciated it was thought best to remove the tooth. After removal it was examined and found the pulp cavity nearly full of ossified material, and the pressure of this upon the seemingly healthy nerve, had produced all the disturbance. In a short time the molar in the other side became very troublesome, and treatment being unsuccessful, it was removed and found to be in the same condition as the previous one. The lady recovered and had no more trouble.

This impingement may be caused by a carbuncle, or boil, and tumors very often cause excruciating pain.

Sailors and ditchers have it more or less, and with this class it is found in the form known as "sciatica" and lumbar neuralgia, more often than in other forms.

One case is reported of a man having neuralgia for six years due to hydrochloric acid absorbed and inhaled by working with it.

And there are cases reported of the shoulder and arm being relieved of severe neuralgic pain by the extraction of a tooth.

Sometimes also we see cases, where the patient has had all the teeth extracted, and still the neuralgia is as bad as ever. A case in point was Mrs. F., of C., age thirty-two, enjoyed good health up to the time of attack; was taken on Saturday afternoon with severe neuralgia of the right side of the face, and having "Dover Powder" in the house she took large doses of that without getting much relief. On Monday she went to the dental office and had all the teeth in that side of the lower maxilla extracted, with no change for the better.

Tuesday a homœopathic practitioner was called who allowed the neuralgia to run its course, but at the end of the week she concluded that something was needed beside time and sugar pellets, so a regular practitioner was called and large doses of morphia and quinine were administered with no better results. Then after the neuralgia had been running for fifteen days I was called, merely for formality, as I afterward learned, as they thought she was going to die, and the friends wanted to give the young practitioner a chance to show his skill, (in making a first class-funeral).

I ordered fl. ext. gelseminum m xv doses every hour, and soon after taking the second dose she turned over and went to sleep, and having slept nearly four hours woke up very much better. I then reduced the dose to m x every two hours until three doses had been taken; after which all medicines were discontinued, and she has not been troubled with neuralgia since.

Whether the gelseminum produced the happy result, or the withdrawal of the medicine, is a question.

Chlorosis is said to be a cause, at least we find chlorotic patients troubled with it more or less. This is probably due to the lowered condition of the vital force.

But in all neuralgias without reference to cause, the most painful points are where the nerve makes its exit from the bone, and it is also a curious fact that in every case we will find the spinous processes of the vertebræ tender on pressure, at a point corresponding, or nearly so, to the point of exit from the intervertebral foramen, and that the pain frequently extends a little further up along the vertebral column.

Thus you see, in patients suffering from "sciatica," pressure on the sacrum ought to give pain, while in patients afflicted with neuralgia of the lumbar plexus, there would be tenderness on pressure of the last dorsal vertebræ.

There is another peculiarity, that is generally overlooked, viz., cutaneous hyperæsthesia at the points of exit of the nerve trunks.

This phenomenon is best studied in cases of inter-costal, lumbar, and crural neuralgia. Its characteristics are such that they can not be mistaken, and may almost be regarded as invariable. When the skin is scratched with the tip of the nail, or gently rubbed with a hard body, as the blunt end of a pencil, the patient complains of a pricking pain, a sensation of burning, which he compares to that felt on rubbing a portion of skin which has been burned to the first degree. Vallix mentions the superficial tender spots:

"The fifth pair of nerves divide into three principal branches, and it is at their points of exit that pain is most particularly felt; namely, over the supra-orbital notch, where the ophthalmic branch becomes superficial, over the infra-orbital foramen, which gives passage to the superior maxillary branch, and over the mental foramen, through which emerges the inferior maxillary division of the nerve. This is easily ascertained by making pressure over these spots with the blunt extremity of a pencil, or with the end of the finger."

A Prossean makes the division into three, with the two principal ones over the spinal processes of the vertebræ, and over the point of peripheral expansion.

Of course these tender spots are good aids in diagnosis; and as such, should be sought after by the profession, as we are working in the dark to a great extent in finding the cause, for cause there is for all the aches and pains that flesh is heir to.

The treatment of neuralgia has been very much bettered in the last few years. For now instead of depletion we have recourse to supportive treatment, and instead of mercury in large doses, we use quinine to eliminate the malarious cachexia that we have in the system, more or less, in this climate.

For neuralgias of the face, of the inferior maxillary in particular, gelseminum seems to have the best effect, how or why I do not pretend to say. Of course every man has his own remedies for such cases.

It has been questioned whether morphia salts that have been used as an hypodermic injection so long and called the "sheet anchor" by our best physicians is the all-powerful remedy it has been supposed to be, as a few experimenters have found that hypodermic injections of cold water produced the same effect that we have supposed morphia produced. Now if water will produce this effect, then why use morphia or any other drug.

Respectfully submitted.

